# Deciphering the Redox-Mediated Interplay Along the Gut–Lung–Brain Axis: Macrophage and Microglia Hyperactivation in Systemic Inflammation

**DOI:** 10.3390/antiox15101342

**Published:** 2026-10-16

**Authors:** Soo-Jin Park, Hee Jae Lee, Su Ui Lee, Jae-Won Lee

**Affiliations:** 1 Natural Product Research Center, Korea Research Institute of Bioscience and Biotechnology, Cheongju 28116, Republic of Korea; soojin921@kribb.re.kr; 2 Department of Pharmacology, College of Medicine, Kangwon National University, Chuncheon 24341, Republic of Korea; heejaelee@kangwon.ac.kr; 3 Immunotherapy Research Center, Korea Research Institute of Bioscience and Biotechnology, Daejeon 34141, Republic of Korea; 4 Department of Biotechnology, University of Science and Technology, Daejeon 34113, Republic of Korea

**Keywords:** gut–lung–brain axis, oxidative stress, microglia-macrophage cross-talk, NLRP3 inflammasome, gasdermin D pore formation, multi-organ-on-a-chip

## Abstract

Emerging paradigms in systemic immunology have illuminated the intricate, bidirectional communication networks linking distant organ systems, which are conceptualized as the gut–lung–brain axis. The homeostasis of this anatomical triad is primarily disrupted by localized and systemic oxidative stress, which serves as a potent driver of chronic inflammatory cascades. This comprehensive narrative review comprehensively delineates the redox-sensitive molecular cross-talk between peripheral tissue-resident macrophages (alveolar and intestinal) and central nervous system microglia. Environmental insults or microbial dysbiosis trigger the overproduction of reactive oxygen species (ROS), which compromise epithelial and endothelial barrier integrity, thereby allowing inflammatory mediators to breach the blood–brain barrier and trigger neuroinflammation. Herein, we systematically evaluate the mechanistic evidence demonstrating how this systemic redox disequilibrium drives coordinated myeloid hyperactivation. Rather than operating as isolated cellular responses, peripheral macrophage activation establishes a self-perpetuating feedback loop that systematically lowers the activation threshold of central microglia. We focus on the molecular convergence of the classical nuclear factor kappa B (NF-κB) pathway and the NOD-like receptor protein 3 (NLRP3) inflammasome within these myeloid lineages, which orchestrates the transcription of cytotoxic cytokines and gasdermin D-mediated pore formation. Crucially, within the architecture of systemic immunology, baseline intracellular ROS production serves as an essential physiological redox signaling molecule requisite for maintaining homeostatic cellular responses, whereas the transition into unmitigated pathological oxidative distress acts as the primary orchestrator of multi-organ barrier collapse. To counteract this multi-organ deterioration, we highlight the translational and therapeutic potential of well-characterized antioxidant phytochemicals—acting as multi-target molecular rheostats capable of neutralizing free radical bursts, rescuing barrier junctions, and systematically reprogramming hyperactive myeloid phenotypes. Furthermore, this review examines the progressive functional impairment and exhaustion of the endogenous cytoprotective Nrf2/HO-1 signaling axis, which functions as the primary molecular rheostat failure under persistent and unmitigated oxidative distress. Finally, we address the strategic implementation of advanced biomimetic multi-organ-on-a-chip technologies designed to model this intercellular dialog, establishing a robust conceptual framework for understanding multi-organ inflammatory decay driven by synchronized macrophage and microglia hyperactivation.

## 1. Introduction

The maintenance of systemic organismal equilibrium relies on a continuous cellular and biomolecular dialog operating across spatial anatomical boundaries, a concept pioneered by modern milestones in translational immunology. Classical physiological paradigms have been used to evaluate the respiratory, gastrointestinal, and central nervous systems as isolated compartmentalized entities governed solely by localized homeostatic mechanisms. However, contemporary evidence has fundamentally dismantled this reductionist view, unveiling an interconnected and highly dynamic multi-directional communicative network designated as the gut–lung–brain axis [1]. Crucially, within the architecture of systemic immunology, reactive oxygen species (ROS) must not be viewed as inherently pathological agents; rather, baseline intracellular ROS production serves as an essential physiological redox signaling molecule requisite for maintaining homeostatic cellular responses. A critical homeostatic threshold exists between this transient, tightly regulated physiological redox signaling and the transition into pathological oxidative distress. While controlled eustress pathways govern cell survival and physiological signaling networks, persistent and unmitigated pathological oxidative distress induces a severe functional impairment of the Nrf2/HO-1 network, transforming ROS into an upstream conductor that fuels runaway multi-organ inflammatory cascades across distinct anatomical barriers [2]. Serving as the primary cellular sentinels within this anatomical triad are specialized tissue-resident mononuclear phagocytes—specifically peripheral alveolar and intestinal macrophages, alongside central nervous system surveillance microglia [3]. These distinct myeloid lineages exhibit extreme sensitivity to circulating reactive oxygen species (ROS) and macromolecular danger signals, undergoing rapid and highly synchronized phenotypic shifts that transform a localized biological insult into a self-perpetuating, multi-systemic pathological cascade [4,5].

The molecular conduit that operationalizes this multi-organ axis is driven by pathologically elevated oxidative stress and compromised structural barriers [6,7]. External environmental insults, notably the inhalation of ambient particulate matter (PM_2.5_) and noxious cigarette smoke, act as localized triggers that initiate volatile ROS generation within the pulmonary epithelium [8]. Interlocking with these respiratory stress signals, gastrointestinal homeostasis is concurrently undermined by a profound disruption of the luminal microenvironment; specifically, a marked reduction in protective short-chain fatty acids (SCFAs) combined with the opportunistic overgrowth of pathobionts accelerates the intraluminal accumulation of cytodestructive free radicals [9]. These localized enteric anomalies prompt an initial oxidative assault on the intestinal mucosa, which fundamentally synergizes with internal systemic challenges; for instance, the translocation of bacterial endotoxins like lipopolysaccharide (LPS) from the compromised lumen into the circulation amplifies this redox imbalance by robustly engaging pattern recognition receptors [10,11]. This converging influx of oxidative insults disrupts the delicate intercellular junctions—such as claudins, occludins, and zonula occludens—that maintain the integrity of both the alveolar–capillary barrier and the intestinal epithelial lining [12]. Once these selective gates are functionally dismantled, a state of hyperpermeability ensues, allowing circulating LPS and localized pro-inflammatory cytokines to breach the systemic vasculature [13]. Ultimately, this biomolecular cascade targets the highly restricted blood–brain barrier (BBB), compromising its structural fidelity and permitting peripheral redox stress to infiltrate the cerebral parenchyma, thereby laying the groundwork for chronic neuroinflammation [14].

At the convergence of this systemic barrier breakdown lies a highly synchronized inflammatory feedback loop orchestrated by myeloid cells via redox-sensitive signaling nodes [15]. Chronic exposure to translocated LPS and circulating ROS shifts both peripheral macrophages and central microglia into a hyper-reactive, pro-inflammatory M1-like state [16]. With respect to the continuum of macrophage behavior, rather than viewing myeloid activation as a simple, rigid binary switch, the phenotypic remodeling of hyperactive myeloid populations operates across a continuous spectrum of cellular states. This natural intervention effectively compresses the expression of classical pro-inflammatory and reactive myeloid states—characterized by classical surface indices specifically cluster of differentiation 80 (CD80) and CD86—alongside signature cytotoxic outputs including iNOS, TNF-α, and ROS bursts. Simultaneously, this homeostatic transition adjusts myeloid immunological tendencies toward a pro-resolving and protective architecture, structurally validated by the restoration of homeostatic microglia signatures and the intensive transcriptional upregulation of specific protective surface receptors, notably CD206 (mannose receptor), alongside cytoplasmic anti-inflammatory counter-regulators including arginase-1 (Arg-1) and chitinase-like 3 (Ym1/Chi3l3). At the intracellular level, this synchronized activation is governed by the classical nuclear factor kappa B (NF-κB) pathway, which upregulates the transcription of canonical cytotoxic mediators, including tumor necrosis factor-alpha (TNF-α) and interleukin-1 beta (IL-1β), interleukin-6 (IL-6), and monocyte chemoattractant protein-1 (MCP-1) [17]. Concurrently, the persistent influx of oxidative stress serves as a secondary priming signal that triggers the assembly of the NOD-like receptor protein 3 (NLRP3) inflammasome complex within both cell types [18]. This redox-dependent molecular machinery drives the enzymatic cleavage and maturation of gasdermin D and pro-inflammatory interleukins, compounding cellular injury and perpetuating a state of chronic cellular alarm [19]. Crucially, this runaway inflammation is further compounded by the concurrent progressive functional impairment and exhaustion of the endogenous cytoprotective nuclear factor erythroid 2-related factor 2 (Nrf2)/heme oxygenase-1 (HO-1) pathway, which fails to sustain systemic redox equilibrium under persistent and unmitigated oxidative distress [20]. Consequently, the reciprocal communication among alveolar macrophages, intestinal macrophages, and cerebral microglia evolves into a self-sustaining pathogenic axis where peripheral oxidative damage directly fuels central neuroinflammatory decay [21,22].

Importantly, this multi-organ communication system is not confined to circulatory and vascular pathways; it is deeply hardwired through parallel autonomic neural routing that operates in tandem with humoral signaling. The vagus nerve functions as a vital bidirectional bridge within the gut–brain–immune triad, capable of rapidly sensing peripheral oxidative threats and translating localized enteric or pulmonary alarm signals directly into the central nervous system. Under physiological or mild acute conditions, this neural connection orchestrates the cholinergic anti-inflammatory pathway—an endogenous reflex loop wherein vagal efferent activation leads to localized acetylcholine release. This neurotransmitter selectively binds to alpha-7 nicotinic acetylcholine receptors (α7nAChR) expressed on macrophages and microglia, acting as a direct biological brake to suppress p-NF-κB translocation and NLRP3 assembly [23,24]. However, during unrelenting systemic redox collapse and massive endotoxemia, this protective neural reflex is overwhelmed. The failure of this autonomic counter-regulatory mechanism dismantles the homeostatic checks on myeloid cells, paving the way for synchronized, unmitigated macrophage and microglia hyperactivation across the entire axis.

However, despite the extensive documentation of single-organ inflammatory pathologies, a critical research gap persists regarding how localized oxidative stress coordinates a synchronized phenotypic polarization cascade across peripheral macrophages and central microglia. Existing literature routinely evaluates these anatomical barriers in isolation, frequently overlooking parallel autonomic neuro-immune pathways—such as the vagal cholinergic reflex governed by acetylcholine and alpha-7 nicotinic acetylcholine receptor (α7nAChR) circuits—and the realistic pharmacokinetic and formulation barriers that dictate the human translation of antioxidant phytochemicals [25]. To address this clinical and mechanistic fragmentation, this review provides a systematic synthesis of the redox-mediated molecular networks governing the gut–lung–brain axis. We first delineate the upstream oxidative cascades and barrier hyperpermeability profiles that operationalize systemic endotoxemia. Next, we evaluate the intracellular signaling nodes—specifically the NF-κB/NLRP3 convergence and the reciprocal progressive impairment of the Nrf2/HO-1 cytoprotective core—that lock myeloid cells into cytotoxic states. Finally, we examine the therapeutic rheostat capacities of representative phytochemical compounds and address the strategic engineering integration of advanced multi-organ-on-a-chip platforms, thereby building a translationally robust framework for multi-organ inflammatory decay.

## 2. Oxidative Stress as a Common Denominator in Gut-Lung-Brain Axis Dysregulation

### 2.1. The Pulmonary-Intestinal Cascade: Localized ROS and Enteric Dysbiosis

The initiation of systemic cross-talk along the gut–lung–brain axis predominantly originates from localized oxidative events within the respiratory architecture. Upon exposure to environmental insults like PM_2.5_ or cigarette smoke, pulmonary epithelial cells and alveolar macrophages demonstrate an immediate redox response, characterized by the hyperactivation of nicotinamide adenine dinucleotide phosphate (NADPH) oxidases and a subsequent surge in intracellular ROS [26]. This localized oxidative strain does not remain confined to the thoracic cavity. Elevated systemic circulation of pulmonary-derived pro-inflammatory cytokines, paired with the direct trafficking of activated immune complexes, alters the homeostatic microenvironment of the gastrointestinal tract [27]. In the enteric lumen, this translated oxidative stress disrupts the delicate ecological balance of the commensal microbiota, inducing microbial dysbiosis characterized by a depletion of beneficial SCFA-producing phyla and pathobiont overgrowth [28]. This microbial shift further exacerbates local free radical generation, converting the gut into a secondary inflammatory epicenter.

Crucially, this interconnected multi-organ framework is not restricted to systemic humoral circulation; it is deeply hardwired through direct neural and specialized metabolite-sensing conduits [29]. The vagus nerve serves as a primary bidirectional neuro-immune bridge within the gut–brain–immune triad, where peripheral nodose ganglia directly detect localized inflammatory bursts [29]. Upon activation, vagal efferent fibers stimulate the cholinergic anti-inflammatory pathway, releasing acetylcholine to bind with α7nAChR expressed on tissue-resident macrophages and microglia, thereby acting as an endogenous neural brake against myeloid hyperactivation [30].

Rather than serving as passive metabolic markers of dysbiosis, microbiota-derived metabolites operate as potent, active upstream signaling ligands that directly govern myeloid cellular states across the axis. Diminished short-chain fatty acid (SCFA) profiles must be understood as a functional withdrawal of critical homeostatic signals, as these metabolites directly ligate transmembrane checkpoints, including free fatty acid receptors 2 and 3 (FFAR2/FFAR3) to dynamically calibrate the activation thresholds and pro-inflammatory or pro-resolving polarization spectrum of intestinal mononuclear phagocytes. Concurrently, aberrant activation of the indoleamine 2,3-dioxygenase 1 (IDO1) enzyme shifts the commensal tryptophan metabolic cascade toward the neurotoxic kynurenine pathway [31]. These hyper-produced kynurenine metabolites act as active systemic signaling vectors that penetrate distinct anatomical barriers to modify microglial surveillance states, demonstrating that microbial metabolites directly dictate systemic immune rheostats rather than merely reflecting ecological fragmentation [31,32].

### 2.2. Epithelial Permeability and Endotoxemia: Breaking the Physical Gates

The compounding oxidative stress generated at both the respiratory and intestinal interfaces directly targets the structural determinants of cellular barrier integrity. Under physiological conditions, the selective permeability of the alveolar–capillary barrier and the intestinal epithelium is rigorously maintained by a protein network comprising claudins, occludins, and zonula occludens (ZO)-1 [33]. Pathologically elevated ROS levels induce the phosphorylation, ubiquitination, and subsequent endocytic degradation of these tight junction proteins [34]. Consequently, this molecular disruption dismantles the cellular gates, giving rise to a phenomenon known as “leaky gut” and compromised lung tissue.

Crucially, this physical barrier breakdown is violently accelerated by specific viral infections, such as severe acute respiratory syndrome coronavirus 2 (SARS-CoV-2) or highly pathogenic influenza strains, which act as secondary biological triggers along the axis [35,36]. These viral pathobionts directly invade alveolar and enteric epithelial monolayers, provoking an abrupt, localized hyperactivation of host NADPH oxidases (NOX) and initiating a robust cellular cytokine storm. This hyper-inflammatory microenvironment works synergistically with baseline environmental oxidative strain to induce the rapid ubiquitination and internalisation of ZO-1 and occludin networks, critically driving the collapse of mucosal structural integrity and worsening macro-molecular systemic leaks [37].

This structural failure permits the systemic translocation of luminal endotoxins, most notably LPS, from the compromised gastrointestinal tract into the portal and systemic vasculature [38]. This state of persistent low-grade endotoxemia serves as a circulating vehicle that propagates macromolecular oxidative signals throughout the systemic circulation, driving the pathology toward more remote organ systems.

### 2.3. Vascular Infiltration and Neuroinflammation: Breaching the Blood–Brain Barrier

The final pathological segment of this axis involves the systematic compromise of the central nervous system’s primary defense line: the BBB. Circulating LPS, alongside elevated systemic ROS and macromolecular cytokines, interacts directly with the brain microvascular endothelial cells (BMECs) forming the capillary walls [39]. This interaction stimulates the endothelial expression of cell adhesion molecules and triggers a localized cascade of free radical generation within the neurovascular unit [40]. The resulting oxidative stress disrupts the specialized tight junctions of the BMECs and induces astrocytic end-foot detachment, functionally opening the structural gates of the BBB. Once the structural fidelity of this barrier is breached, peripheral redox stress and circulating endotoxins infiltrate the cerebral parenchyma. This cerebral invasion immediately engages the toll-like receptor 4 (TLR4) complexes on surveillance microglia, initiating a self-perpetuating neuroinflammatory response that underscores chronic neurodegenerative progression [41]. To provide a cohesive overview of these tissue-specific defects, the distinct external triggers, compromised barrier networks, and primary intracellular alarm signals characterizing each anatomical segment are systematically stratified in Table 1.

## 3. Cross-Talk Between Macrophages and Microglia via Redox Signaling

### 3.1. Phenotypic Synchronization: The Pro-Inflammatory Myeloid Spectrum

The systemic escalation of oxidative stress serves as a synchronized phenotypic switch that shifts both peripheral macrophages and central nervous system microglia away from homeostatic surveillance toward hyper-reactive, pro-inflammatory and reactive myeloid states [42]. Upon detecting circulating LPS and transient ROS, TLR4 complexes on alveolar and intestinal macrophages, as well as cortical microglia trigger a cascade of intracellular phosphorylation events [43].

Mechanistically, this pattern recognition receptor targets a strictly bifurcated downstream signaling network consisting of the canonical MyD88-dependent pathway and the parallel MyD88-independent (TRIF-dependent) conduit [44]. The early-phase inflammatory response is predominantly operationalized by the MyD88-dependent arm, which recruits interleukin-1 receptor-associated kinases (IRAKs) to activate the IκB kinase (IKK) complex, thereby catalyzing the rapid nuclear translocation of the NF-κB p65 subunit [44]. Concurrently, the late-phase inflammatory network is governed by the endosomal TRIF-dependent track, which coordinates TANK-binding kinase 1 (TBK1) activation to actively phosphorylate interferon regulatory factor 3 (IRF3) [45]. This independent signaling axis drives the nuclear entry of IRF3 to command the sustained transcription of Type I interferons (IFN-α/β) and delayed wave cytotoxic mediators, ensuring persistent myeloid vigilance.

**Figure 1:**
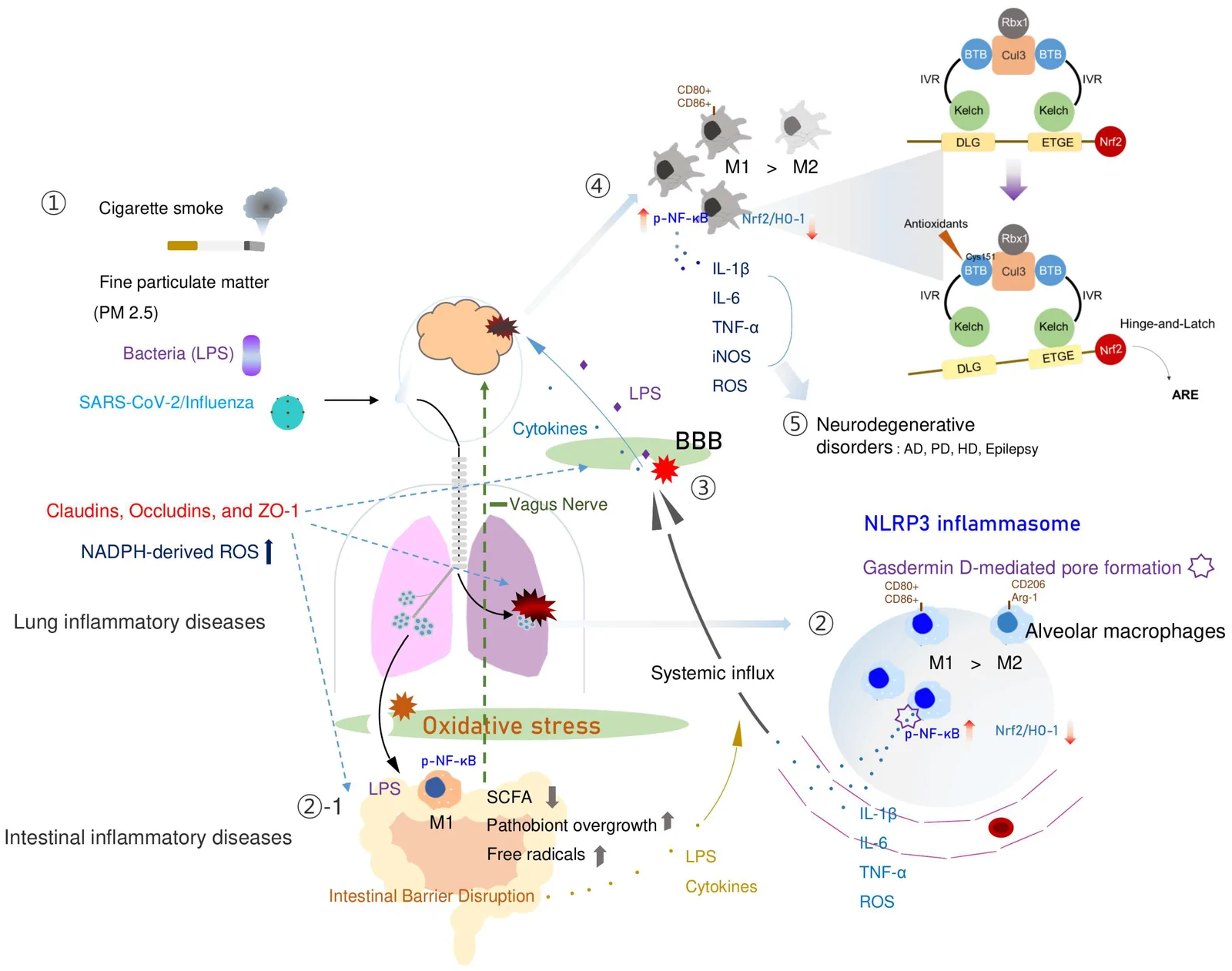
Schematic diagram of the redox-mediated pathophysiological cascade along the gut–lung–brain axis and the molecular failure of cellular rheostats during macrophage and microglia hyperactivation. ① The breakdown of the systemic interconnectedness of the anatomical triad is initiated by localized external environmental insults—such as inhaled ambient particulate matter (PM_2.5_) and noxious cigarette smoke—within the respiratory architecture, alongside internal endotoxemic challenges (lipopolysaccharide, LPS) driven by enteric microbial dysbiosis. These converging oxidative insults trigger the hyperactivation of localized NADPH oxidases, driving a massive surge in intracellular reactive oxygen species (ROS). Consequently, this localized redox strain causes the phosphorylation and subsequent endocytic degradation of essential tight junction networks (claudins, occludins, and ZO-1), dismantling the structural fidelity of both the alveolar–capillary barrier and the intestinal epithelial lining. ②, ②-1 The ensuing hyperpermeability allows the systemic translocation of circulating LPS, diminished short-chain fatty acids (SCFAs), and macromolecular cytokines into the vasculature. This systemic influx shifts peripheral tissue-resident myeloid lineages—specifically alveolar macrophages (②) and intestinal macrophages (②-1)—into hyper-reactive, pro-inflammatory and reactive myeloid states governed by p-NF-κB activation and NLRP3 inflammasome-mediated gasdermin D pore formation. ③ These circulating inflammatory mediators ultimately target and breach the highly restricted BBB via neurovascular disruption. ④ Upon gaining entry into the cerebral parenchyma, peripheral redox signals induce a synchronized phenotypic polarization in central surveillance microglia. This triggers an intensive reactive myeloid hyperactivation that accelerates the transcription of neurotoxic components (IL-1β, IL-6, TNF-α, iNOS, and ROS). Concurrently, the endogenous vagal cholinergic anti-inflammatory pathway—which normally utilizes acetylcholine and alpha-7 nicotinic receptors (α7nAChR) to act as a direct neural brake against this central and peripheral myeloid hyperactivation—becomes functionally overwhelmed and dismantled. ⑤ Crucially, this homeostatic failure is driven by the progressive functional impairment and exhaustion of the endogenous Keap1-Nrf2/ARE defense core, a molecular rheostat failure operating universally within both central surveillance microglia and peripheral alveolar/intestinal macrophages [20,48,49]. Under unrelenting systemic oxidative stress, the physical docking between Keap1 domains and the Cul3/Rbx1 ubiquitin ligase framework targets newly translated Nrf2 for continuous proteasomal destruction, stripping both myeloid lineages of their cellular buffer systems.

This molecular signal drives canonical reactive myeloid hyperactivation, characterized by the marked upregulation of inducible nitric oxide synthase (iNOS) and cyclooxygenase-2 (COX-2) [46]. Rather than operating as isolated cellular responses, the peripheral activation of macrophages prompts a reciprocal wave of microglial reactivity. This intercellular synchronization establishes a self-perpetuating, multi-organ feed-forward loop, where peripheral free radical production systematically lowers the threshold for microglial hyperactivation, thereby magnifying the susceptibility of the cerebral parenchyma to chronic inflammation [47]. This comprehensive roadmap of redox-mediated tissue-to-tissue cross-talk and progressive systemic barrier failure along the gut–lung–brain cascade is schematically illustrated in Figure 1.

To counteract this multi-organ decay, theoretical or preclinical implementation of electrophilic or polyphenolic antioxidants—most notably sulforaphane and resveratrol—has been empirically proven in established in vitro myeloid models (RAW 264.7, BV2) and in vivo animal profiles to revive this cytoprotective axis [48,50,51,52,53]. Mechanistically, sulforaphane covalently modifies the Keap1 Cys151 residue to freeze the Cul3 ligase machinery, while resveratrol competitively intercepts the spatial binding between the KEAP1 Kelch domain and the NRF2-DLG motif interface [53]. These precise conformational updates selectively trigger the “Hinge-and-Latch” dissociation at the DLG/ETGE motif junctions, successfully rescuing Nrf2 from ligase capture and permitting its nuclear translocation to bind onto the Antioxidant Response Element (ARE) to fuel massive HO-1 and SOD transcription [48]. As established preclinical channels, this core engine activation universally dampens hyper-reactive cascades by suppressing downstream iNOS/ROS production in alveolar myeloid lines [54], while simultaneously rescuing cellular function by enhancing the defective opsonic phagocytosis and bacterial clearance capacity of compromised mucosal macrophages [51]. Conversely, as a targeted future translational framework, while these compounds have successfully transitioned into human Phase I/II clinical trials demonstrating potent systemic antioxidant biomarker improvements and safety, their actual translational utility remains strictly constrained by poor aqueous solubility and intense first-pass biotransformation, highlighting the prerequisite for next-generation nano-delivery matrices to sustain functional therapeutic levels within the multi-organ triad [55,56].

### 3.2. Intracellular Signaling Nodes: The Convergence of NF-κB and the NLRP3 Inflammasome

At the center of this redox-mediated cellular alarm are two converging intracellular signaling axes: the nuclear factor kappa B (NF-κB) pathway and the NLRP3 inflammasome [57]. Intracellular ROS accumulation acts as an obligate secondary messenger that promotes the phosphorylation and subsequent proteasomal degradation of the inhibitory subunit IκBα, thereby liberating the NF-κB p65 subunit to translocate into the nucleus [58]. This transcriptional event drives the de novo synthesis of pro-inflammatory cytokines such as TNF-α and pro-IL-1β. Simultaneously, this oxidative microenvironment acts as the definitive priming and activation signal for the assembly of the NLRP3 inflammasome complex [59]. The physical maturation of this multiprotein platform activates caspase-1, which enzymatically cleaves gasdermin D to form membrane pores, facilitating the unconventional secretion of mature IL-1β and IL-18. This dual-pathway convergence transforms both macrophages and microglia into potent cellular micro-factories of cytotoxic mediators, converting localized redox imbalance into systemic tissue injury.

Crucially, this hyper-inflammatory orchestration is molecularly locked into a strict inverse relationship with the cell’s internal protective machinery, functioning as a reciprocal negative feedback loop where the prompt mobilization of NF-κB/NLRP3 complexes directly forces the functional shutdown of counter-regulatory networks [60,61]. Under unrelenting cellular strain, the mechanical expansion of these pro-inflammatory cascades works dynamically to dismantle the activation thresholds of homeostatic response engines, transitioning the pathology from localized warning responses into systemic adaptive failures.

### 3.3. Collapse of Endogenous Counter-Regulators: Nrf2/HO-1 Signaling Failure

Under substantial oxidative stress, the critical determinant of chronic pathology is the progressive functional impairment of the endogenous cytoprotective mechanisms governed by the Nrf2 signaling pathway [26]. Under basal physiological conditions, Nrf2 is constitutively sequestered in the cytoplasm by Kelch-like ECH-associated protein 1 (Keap1) for proteasomal degradation. Although transient elevations in ROS typically stimulate Nrf2 stabilization and nuclear translocation to bind with antioxidant response elements (AREs), persistent and unmitigated oxidative insults induce a severe functional impairment and progressive exhaustion of this cytoprotective network, rather than a total molecular collapse [62]. The functional suppression of this endogenous antioxidant machinery prevents the adequate transcription of phase II detoxifying enzymes, most notably, heme oxygenase-1 (HO-1) and superoxide dismutase (SOD). The structural strain on this molecular rheostat leaves both peripheral macrophages and central microglia with attenuated homeostatic thresholds, heavily suppressing the cell’s ability to balance NF-κB-driven transcription and NLRP3 assembly, thereby lock the gut–lung–brain axis into a chronic inflammatory state.

## 4. Antioxidant Phytochemicals as Multi-Target Rheostats

### 4.1. Structural Diversity of Phytochemical Taxonomy and Molecular Versatility

The structural heterogeneity inherent in botanical secondary metabolites—predominantly classified into polyphenols, flavonoids, alkaloids, and terpenoids—provides a unique biochemical platform for multi-target pharmacological interventions along the gut–lung–brain axis [63]. Classical drug discovery paradigms historically prioritized isolated monocultures designed to target single, discrete receptors; however, such monolithic approaches routinely fail to resolve complex, systemic disorders driven by multi-organ axis dysregulation. In contrast, antioxidant phytochemicals possess dense arrays of hydroxyl groups, aromatic rings, and electrophilic centers that confer structural versatility. At the systemic level, these diverse chemical configurations enable natural formulations to simultaneously interact with multiple distinct intracellular networks. Rather than acting as simple chemical agents, these naturally occurring complexes exhibit sufficient lipophilic and hydrophilic latency to retain structural stability across biological boundaries, offering a conceptual framework for synchronous cellular protection across remote tissue systems [64].

### 4.2. Theoretical Reprogramming of Myeloid Lineages: Modulating the M1/M2 Phenotypic Balance

Within the framework of systemic inflammation, structurally diverse phytochemical classes offer a compelling theoretical strategy for driving the precise cellular reprogramming of tissue-resident mononuclear phagocytes. Rather than viewing myeloid activation as a simple, rigid binary switch, the phenotypic remodeling of hyperactive myeloid populations operates across a continuous spectrum of cellular states. In both peripheral alveolar/intestinal macrophages and central nervous system microglia, specific flavone aglycones and isoquinoline alkaloid cores possess the structural configuration required to intercept upstream pattern recognition receptors. Mechanistically, this chemical interference blunts the phosphorylation cascades that liberate NF-κB p65 subunit, thereby halting its nuclear translocation and subsequently dismantling the downstream synthesis of canonical cytotoxic cytokines [65]. Simultaneously, these biochemical modulators interrupt the mitochondrial secondary priming phases essential for the spatial assembly of the NLRP3 inflammasome complex. By compressing these classical pro-inflammatory and reactive signaling nodes, the natural intervention of antioxidant compounds adjusts myeloid immunological tendencies toward a pro-resolving and protective architecture, characterized by the sustained transcription of cytoprotective markers such as arginase-1 (Arg-1) and interleukin-10 (IL-10) [66].

To ground these paradigms into verifiable pharmacology, specific natural polyphenols, flavonoids, and alkaloids offer precise multi-target efficacy instead of operating as vague chemical categories [67,68,69]. This natural intervention drives the precise phenotypic remodeling of hyperactive myeloid populations, effectively compressing the expression of classical pro-inflammatory and reactive myeloid states—characterized by classical surface indices specifically cluster of differentiation 80 (CD80) and CD86—alongside signature cytotoxic outputs including iNOS, TNF-α, and IL-6 [68,69]. For instance, curcumin and quercetin chemically interfere with the IκBα kinase complex, arresting NF-κB p65 nuclear entry and suppressing upstream NLRP3 inflammasome priming phases [67,69]. Simultaneously, berberine and epigallocatechin gallate (EGCG) suppress these pro-inflammatory and reactive signaling nodes, resetting myeloid immunological tendencies toward a homeostatic and pro-resolving spectrum [69]. This homeostatic transition is molecularly validated by the intensive transcriptional upregulation of specific protective surface receptors, notably CD206 (mannose receptor), alongside cytoplasmic anti-inflammatory counter-regulators including Arg-1, chitinase-like 3 (Ym1/Chi3l3), and the protective cytokine IL-10 [70].

### 4.3. Natural Induction of the Nrf2/HO-1 Core Engine: Restoring Systemic Redox Equilibrium

The definitive molecular mechanism by which plant-derived secondary metabolites can conceptually counteract gut–lung–brain axis disruption is through the direct pharmacological stabilization of the endogenous Nrf2/HO-1 cytoprotective signaling pathway. Under baseline physiological status, Nrf2 remains constitutively bound in the cytoplasm by Kelch-like ECH-associated protein 1 (Keap1), which targets it for continuous proteasomal clearance. Although transient elevations in ROS typically stimulate Nrf2 stabilization and its subsequent nuclear translocation to interact with antioxidant response elements (AREs), persistent and unmitigated oxidative insults induce a progressive functional impairment and exhaustion of this cytoprotective network, rather than a total molecular collapse [49].

Bioactive phytochemical structures, particularly those bearing specialized electrophilic moieties such as sulforaphane and resveratrol, are uniquely capable of chemically modifying specific, highly reactive cysteine residues (specifically Cys151) on the sensor protein Keap1 [71]. Structurally, Keap1 operates as a homodimeric substrate adaptor comprising a Broad-Complex, Tramtrack, and Bric-a-brac (BTB) domain, an intervening Inter-Light Chain/Inter-Variable Region (IVR), and a C-terminal Kelch domain that anchors the Nrf2 molecule. Under baseline conditions, the IVR and BTB domains physically coordinate with the scaffold protein Cullin 3 (Cul3) and Ring Box Protein 1 (Rbx1) to assemble a functional E3 ubiquitin ligase complex [72].

When active electrophilic natural compounds introduce covalent modifications directly onto the highly sensitive Cys151 residue situated within the BTB domain, it induces a steric and conformational rearrangement across the Keap1 dimer interface. This precise molecular shift breaks the spatial docking configuration and physical interaction between the IVR domain and the Cul3-ubiquitin ligase framework, effectively freezing the polyubiquitination machinery. Parallelly, distinctive natural polyphenols, most notably resveratrol, stabilize this protective pathway through an alternative non-covalent interface, directly targeting the NRF2-DLG motif to competitively disrupt its physical structural binding with the KEAP1 Kelch domain. Consequently, through these bifurcated protective modes, newly translated Nrf2 entities successfully evade continuous proteasomal destruction [73] and translocate unimpeded into the nucleus. Upon stabilizing and entering the nucleus, Nrf2 binds directly to AREs located within the promoter domains of cytoprotective target genes to coordinate the robust transcription of a broad array of phase II detoxifying enzymes, most notably HO-1 and SOD [74].

The conceptual implementation of natural Nrf2 induction thus provides a sustainable, multi-systemic mechanism capable of mitigating runaway intracellular ROS bursts, balancing iNOS and COX-2 enzymatic outputs, and systematically supporting the restoration of systemic redox equilibrium across distinct anatomical barriers.

However, the translational implementation of these compounds is fundamentally constrained by their intrinsic pharmacokinetic realities [75]. While alkaloids like berberine display a moderate capacity to breach the highly restricted BBB via active transporters [76], hydrophobic polyphenols such as resveratrol and EGCG suffer from intense intestinal first-pass metabolism and rapid systemic clearance [77]. This results in poor systemic bioavailability that necessitates advanced delivery strategies to achieve therapeutic levels within the central nervous system [78]. To provide a comprehensive overview of these experimental validations and pharmacological behaviors, the structural classification, target mechanisms, and pharmacokinetic profiles of these well-characterized agents are systematically stratified in Table 2.

## 5. Present Limitations and Strategic Future Models

### 5.1. Methodological Fragmentations: The Critical Absence of Integrated Multi-Organ Models

The main challenge that currently impedes the validation of the gut–lung–brain corridor is the notable lack of standardized, integrated experimental models that can simultaneously capture all segments of this anatomical triad. Currently, multi-systemic investigative research relies heavily on disconnected, compartmentalized testing protocols, such as evaluating an oxidative insult or cell signaling cascade exclusively on an isolated pulmonary cell line or a singular enteric microenvironment. This methodological isolation fails to truly replicate the physiology of systemic immunological cross-talk. Demonstrating that a localized stimulus modulates macrophage activation in the respiratory tract does not scientifically prove its downstream impact on microglial hyperactivation, as the intervening vascular, metabolic, and microbial transformations are completely overlooked. Until the field transitions away from these reductionist individual models toward validated, interconnected multi-organ platforms, predicting the precise holistic impact of multi-organ inflammatory decay will remain an ongoing challenge.

### 5.2. Advanced Preclinical Platforms: Multi-Organ-on-a-Chip Systems

To overcome the inherent limitations of fragmented monocultures, the implementation of multi-organ-on-a-chip (MOC) platforms has emerged as a critical biotechnological imperative. These microfluidic systems mechanically replicate the dynamic fluid shear stress, vascular perfusion, and complex tissue–tissue interfaces characterizing human systemic physiology [119]. A synchronized lung–gut–brain chip conceptually integrates human alveolar epithelial channels, intestinal epithelial monolayers, and specialized BBB circuits containing brain microvascular endothelial cells, astrocytes, and surveillance microglia [120]. By interconnecting the perfusion of these distinct compartmentalized chambers, investigators can introduce localized environmental insults—such as aerosolized PM_2.5_ or localized endotoxins—to the respiratory or enteric interfaces. This biomimetic approach enables precise micro-spatial tracking of translocated biomarkers, mapping how peripheral redox stress propagates across distinct anatomical boundaries to trigger neuroinflammatory microglial activation in real time.

### 5.3. Next-Generation Architectural Paradigms: Three-Dimensional Organoids and Microfluidic Integration

To fully capture the intricate cellular heterogeneity and spatial architecture of individual organ barriers, the integration of three-dimensional (3D) organoids represents a groundbreaking preclinical advancement. Patient-derived or induced pluripotent stem cell (iPSC)-derived pulmonary, intestinal, and cerebral organoids closely represent the spatial orientation, extra-cellular matrix deposition, and physiological secretome of in vivo native tissues [121]. When integrated within microfluidic architectures, these 3D models facilitate a more accurate evaluation of systemic cell-to-cell communication networks. Unlike flat 2D monolayers, integrated organoid systems permit the rigorous study of complex systemic challenges, enabling the assessment of multi-target intercellular feedback loops, systemic metabolic transformations, and subsequent signal penetration through the BBB [119]. This humanized cellular framework allows researchers to investigate whether intrinsic antioxidant defenses preserve tight junction networks and modulate myeloid polarization within a highly architectural, multi-systemic environment.

### 5.4. Unresolved Technical Bottlenecks: Engineering and Biological Limitations of Advanced Preclinical Models

Despite the transformative potential of biomimetic MOC arrays and three-dimensional organoid architectures, several critical technological hurdles must be resolved before their widespread translational adoption [122]. A primary engineering limitation is the non-specific hydrophobic absorption of lipophilic bioactive agents and natural phytochemicals by polydimethylsiloxane (PDMS), the elastomer most widely used to fabricate microfluidic channels [123]. This material sequestration artificially lowers the active concentration of compounds within the media perfusion circuits, introducing significant experimental bias during pharmacological and pharmacokinetic evaluations [123].

Biologically, current organoid and MOC configurations lack a functional adaptive immune system, operating almost exclusively with isolated innate myeloid lineages (macrophages and microglia) without the critical systemic integration of exogenous T-cells, B-cells, or functional circulating lymphatics [124]. Finally, these complex advanced platforms face immense challenges regarding standardization, batch-to-batch structural reproducibility, and high fabrication costs [125]. These manufacturing and operational complexities restrict their compatibility with large-scale high-throughput screening applications, highlighting the urgent engineering and economic refinements required to bridge the gap between preclinical discovery and clinical translation.

## 6. Conclusions

The historical medical dogma of evaluating and treating respiratory, gastrointestinal, and neurological disorders as isolated, compartmentalized pathological events is fundamentally incompatible with the emerging principles of systemic translational immunology. In this comprehensive narrative review, we comprehensively explored how the gut–lung–brain axis operates as a highly dynamic, redox-sensitive communicative highway where localized environmental and internal endotoxemic insults trigger a destructive, multi-organ inflammatory cascade. The synchronized hyperactivation of tissue-resident mononuclear phagocytes—specifically peripheral alveolar/intestinal macrophages and central surveillance microglia—represents the definitive unifying pathological hallmark of this axis. This cellular interconnection demonstrates that chronic tissue decay is not a localized event but rather a coordinated, multi-systemic phenomenon driven by barrier failure and runaway intracellular oxidative stress that causes a progressive functional impairment and exhaustion of endogenous cytoprotective rheostats.

Crucially, this bidirectional communication network is structurally integrated not only via vascular and humoral leakage pathways but also through direct autonomic neuro-immune circuits, such as the vagal cholinergic anti-inflammatory pathway, alongside specialized microbiota-derived tryptophan and metabolite-sensing matrices. Furthermore, while antioxidant phytochemicals offer a promising multi-target strategy to reprogram these hyperactive myeloid lineages and revive the Nrf2/HO-1 defense core, their ultimate clinical translation remains strictly determined by key pharmacokinetic parameters, including gastrointestinal biotransformation and blood–brain barrier penetration efficiencies.

While the current absence of unified, multi-organ experimental platforms poses a legitimate challenge to immediate preclinical validation, acknowledging these methodological fragmentations provides a transparent roadmap for future investigative frameworks. Shifting the investigative paradigm away from reductionist individual monocultures toward biomimetic MOC technologies and three-dimensional organoid co-culture circuits will allow researchers in this field to decode these intercellular dialog networks with unprecedented spatial and temporal resolution. Looking forward, the upcoming decade is projected to witness the strategic translation of these bio-inspired platforms into actionable human clinical applications. By marrying targeted antioxidant nano-formulations with patient-derived multi-organ diagnostic frameworks, the field will transition toward non-invasive, patient-specific precision medicine pipelines. This translational trajectory will empower clinicians to screen and personalize multi-target phytochemical formulations, establishing a protective rheostat that seals intestinal leaks, calms pulmonary oxidative bursts, and halts microglial hyperactivation before neurological deterioration becomes irreversible. Ultimately, capturing the molecular convergence of redox-sensitive pathways and myeloid phenotypic synchronization establishes a substantial conceptual foundation, guiding future translational strategies to effectively restore systemic homeostasis and safeguard human physiological integrity across the gut–lung–brain axis.

## Figures and Tables

**Table 1:** Comprehensive stratification of redox-dependent signaling pathways and barrier malfunctions across the gut–lung–brain axis.

Anatomical Triad	Primary Inducers	Myeloid Lineages & Phenotypes	Targeted Barrier Components	Key Intracellular Signaling Nodes	Molecular Dysregulations & Pathological Indicators	Potential Therapeutic Intervention Targets & Evidence Models
Respiratory System (Lung)	Inhalation of PM2.5, tobacco smoke exposure, viral pathogens (SARS-CoV-2/Influenza)	Alveolar macrophages (CD80+, CD86+ pro- inflammatory M1 polarization)	Claudins, occludins, and ZO-1 networks (Alveolar- capillary barrier)	Activation of NADPH oxidase complexes, bifurcated TLR4 downstream (MyD88 and endosomal TRIF matrices), NF-κB p65 nuclear entry, iNOS induction	Over-activation of NOX2/NOX4, endocytic internalization of ZO-1, viral-induced cytokine storm, elevated pulmonary ROS output	• Targets: NOX2/NOX4 enzymatic inhibitors, TLR4/MyD88/TRIF pathway blockers, NF-κB p65 phosphorylation arrestors • Evidence Level/Models: Validated in vitro (A549, primary alveolar macrophages) and in vivo (LPS/viral-induced murine acute lung injury models)
Gastrointestinal Tract (Gut)	Systemic pulmonary cytokines, microbial dysbiosis, luminal pathobiont infiltration	Intestinal macrophages (CD80+, CD86+ reactive M1 phenotype)	Occludin and ZO-1 Proteins (Intestinal Epithelial border	Mucosal ROS accumulation, diminished SCFA levels, RAGE/p38 MAPK core activation, enteric MLCK induction	Loss of SCFA- Synthesizing phyla, aberrant IDO1 activation (kynurenine pathway shift), ubiquitination of tight junctions, systemic endotoxemia via LPS trans-location	• Targets: MLCK expression suppressors, IDO1 metabolic inhibitors, specialized metabolite-sensing receptor (FFAR2/FFAR3) agonists • Evidence Level/Models: Substantiated in vitro (Caco-2 monolayers, RAW 264.7) and in vivo (DSS-induced colitis and microflora depletion animal arrays)
Central Nervous System (Brain)	Vascular LPS infiltration, systemic ROS/cytokines, peripheral macrophage spillover	Surveillance microglia (CD80+, CD86+ hyper-reactive M1 polarization)	BMEC tight junctions, disruption of astrocytic end-feet	NLRP3 Inflammasome scaffolding, caspase-1 activation, gasdermin D proteolysis, IRF3 phosphorylation (Type I IFNs synthesis)	Functional exhaustion of Nrf2/HO-1 cascade, gasdermin D-driven transmembrane pore assembly, vagal cholinergic reflex failure (α7nAChR breakdown), neurodegenerative progression	• Targets: NLRP3 inflammasome assembly inhibitors, Gasdermin D pore blockers, Nrf2 stabilizing electrophiles, alpha-7 nicotinic receptor (α7nAChR) systemic agonists • Evidence Level/Models: Demonstrated in vitro (BV2, primary microglia) and in vivo (neurodegenerative, stroke, and vagotomy challenge murine models)

Abbreviations: α7nAChR, alpha-7 nicotinic acetylcholine receptor; CD80/CD86, cluster of differentiation 80/86; CD206, cluster of differentiation 206 (mannose receptor); CNS, central nervous system; FFAR2/FFAR3, free fatty acid receptor 2/3; IDO1, indoleamine 2,3-dioxygenase 1; iNOS, inducible nitric oxide synthase; IRF3, interferon regulatory factor 3; LPS, lipopolysaccharide; MLCK, myosin light chain kinase; NADPH, nicotinamide adenine dinucleotide phosphate; NF-κB, nuclear factor kappa B; NOX, NADPH oxidase; NLRP3, NOD-like receptor protein 3; PM 2.5, particulate matter 2.5; RAGE, receptor for advanced glycation endproducts; ROS, reactive oxygen species; SCFAs, short-chain fatty acids; TRIF, TIR-domain-containing adapter-inducing interferon-β; Ym1, chitinase-like 3; ZO-1, zonula occludens-1.

**Table 2:** Pharmacological stratification, molecular targets, and pharmacokinetic profiles of well-characterized antioxidant phytochemicals along the gut–lung–brain axis.

Phytochemical Compound/ Reference	Chemical Class	Experimental Model	Specific Molecular Targets & Mechanisms	Pharmacokinetic Parameters & Bioavailability Realities
Curcumin [79,80,81,82,83,84]	Polyphenolic Curcuminoid	In vitro (RAW 264.7, BV2 & Caco-2/HT-29); In vivo (Murine LPS & High-Fat Diet models); In vivo gut microbiome profiles	Suppresses NLRP3 inflammasome assembly; blocks NF-κB p65 nuclear entry while simultaneously activating the Nrf2/HO-1 axis to upregulate NQO1 and GST; promotes protective, pro-resolving myeloid states. Inhibits p38 MAPK phosphorylation and MLCK expression to prevent tight junction (ZO-1, claudin-1/7) degradation; increases beneficial taxa (*Akkermansia*, *Lachnospiraceae*) while downregulating enteric IL-1β.	Extremely poor Intestinal bioavailability (<1%); restricted baseline BBB penetration due to rapid systemic glucuronidation. Orally administered fractions accumulate heavily within the gastrointestinal tract, enabling direct luminal interaction with enterocytes and local microbial niches despite minimal systemic tissue distribution.
Sulforaphane [48,55,85,86,87,88,89,90,91]	Isothiocyanate (Organosulfur)	In vitro (Alveolar epithelial cells, macrophages & Caco-2 monolayers); In vivo (Neuroinflammation & DSS-induced colitis models); In vivo cecum/colon microflora arrays	Covalently Modifies Keap1 (Cys151) to disrupt the “hinge-and-latch” interaction, driving intensive Nrf2 nuclear escape; upregulates HO-1, SOD, and CAT. Mitigates TNFα-induced enterocyte ROS/H2O2 production; downregulates NOX1/NOX4 expression; preserves epithelial thickness by enriching butyrate-producing probiotics (e.g., *Butyricicoccus*).	High Gastrointestinal absorption and rapid bioavailability; effectively crosses the BBB to exert central cytoprotection. Readily absorbed and metabolized inside Caco-2 enterocytes via glutathione conjugation; unabsorbed precursors interact with extracellular transmembrane receptor domains to maintain intestinal redox balance.
Resveratrol [53,92,93,94,95,96,97,98]	Stilbenoid (Polyphenol)	In vitro (RAW 264.7, BV2 & Caco-2); In vivo (Neurodegenerative & IBD models); In vitro gut microbiota fermentation	Activates SIRT1 to deacetylate and suppress NF-κB p65 and NLRP3 inflammasome assembly; concurrently upregulates the Nrf2/HO-1 signaling cascade; shifts pro-inflammatory and reactive myeloid states toward homeostatic spectrums. Directly targets the NRF2-DLG motif interface to competitively disrupt its structural physical binding with the KEAP1 Kelch domain, evading proteasomal clearance; Upregulates tight junction (ZO-1, occludin) and mucin mRNA expressions; suppresses enteric NF-κB cascades; reshapes commensal microenvironment by promoting protective phyla (e.g., *Lactobacillus reuteri*).	Rapid intestinal absorption but extremely low systemic bioavailability (<5%) due to extensive phase II metabolism; transient BBB crossing. Undergoes intense intestinal biotransformation into sulfation/glucuronidation metabolites; active metabolites accumulate substantially within the intestinal tract and feces to drive localized gut barrier restoration.
Quercetin [99,100,101,102,103]	Flavonol (Flavovoid)	In vitro (RAW 264.7, Alveolar epithelial A549 cell lines & Caco-2), In vivo (neuroinflammation & DSS colitis murine models)	Inhibits NLRP3 inflammasome activation; stimulates Nrf2-mediated antioxidant defenses and modulates PKC signaling to preserve tight junction networks; reduces TNF-α and IL-6 expression. Halts indoleamine 2,3-dioxygenase 1 (IDO1) activation in both colon and brain to block the neurotoxic kynurenine pathway; intercepts the RAGE/NF-κB core to suppress lung and hypothalamic hyperinflammation.	Low aqueous solubility and poor oral bioavailability (~1%); metabolites display limited and slow penetration across the BBB. Subject to extensive bioconversion by commensal microflora into smaller phenolic acids, which stimulate SCFA-producing phyla (e.g., *Dubosiella*) to reinforce systemic barrier integrity.
Berberine [104,105,106,107,108,109,110]	Isoquinoline Alkaloid	In vitro (BV2 & primary microglia/macrophages); Ex vivo (Intestinal organoid-macrophage co-cultures); In vivo (Stroke & IBD animal models)	Activates AMPK to dampen downstream TLR4/NF-κB signaling loops; prevents gasdermin D-mediated pore formation; mitigates enteric endotoxemia. Suppresses CXCL1 and macrophage migration inhibitory factor (MIF) from epithelial blocks to disrupt organoid-myeloid recruitment; blocks p38 MAPK and IKKβ cascades to skew microglia toward homeostatic, pro-resolving spectrums.	Low intestinal absorption rate (~5%) due to P-glycoprotein efflux; moderately breaches the BBB via active transport matrices. Exerts high biological actions locally on the gut mucosa before systemic degradation; shifts bile acid pools and fecal metabolite tracking (SCFAs/TMAO) to decrease lipopolysaccharide-binding protein
Epigallocatechin gallate (EGCG) [111,112,113,114,115,116,117,118]	Flavan-3-ol (Catechin)	In vitro (Myeloid lines, Caco-2 monolayers, & pulmonary cell grids); In vivo (AD/PD & Acute Respiratory Distress Syndrome, ARDS models)	Scavenges intracellular ROS directly; coordinates the phenotypic switch of hyperactivated, reactive myeloid cells toward CD206-expressing pro-resolving architectures by suppressing p-NF-κB and NADPH oxidase (NOX) overactivation. Activates the intestinal AhR pathway via microbial indole-derivative induction to fortify gut physical locks; downregulates NOX-driven oxidative bursts to attenuate pulmonary immune cell infiltration.	Low oral absorption and low systemic availability (<1%); limited baseline BBB permeability, requiring high systemic dosing. Suffers from rapid intestinal first-pass metabolism (glucuronidation/methylation); extensively biotransformed in the distal colon into active bioavailable gallic acid derivatives to secure systemic shields.

## Data Availability

No new data were created or analyzed in this study. Data sharing is not applicable to this article.
